# Severe hypertriglyceridemia secondary to venlafaxine use in an older adult on dialysis -case report

**DOI:** 10.1186/s12913-017-2195-2

**Published:** 2017-04-13

**Authors:** Hsiang-Wen Lin, Cory A. Simonavice, Chiung-Ray Lu, Wen-Ling Lin, Po-Lun Wu, Che-Yi Chou, Chun-Hui Liao, Hsieh-Yuan Lane

**Affiliations:** 1 School of Pharmacy and Graduate Institute, College of Pharmacy, China Medical University, No. 91 Hsueh-Shih Road, Taichung, 40402 Taiwan; 2 Department of Pharmacy, China Medical University Hospital, No. 2 Yuh-Der Road, Taichung, 40447 Taiwan; 3 Department of Internal Medicine, China Medical University Hospital, No. 2 Yuh-Der Road, Taichung, 40447 Taiwan; 4 Department of Psychiatry, China Medical University Hospital, No. 2 Yuh-Der Road, Taichung, 40447 Taiwan

**Keywords:** Hypertriglyceridemia, Hemodialysis, Venlafaxine, Older Adult, Case report

## Abstract

**Background:**

Although the prescribing information for Venlafaxine extended release includes a discussion about possible increases in total cholesterol and triglycerides (TG) seen in healthier adult patients during premarketing clinical trials, no post-marketing studies or case reports, that discuss the effects of venlafaxine on TG in elderly patients with chronic kidney disease.

**Case presentation:**

We report a 71 year-old male patient with end-stage renal disease on hemodialysis, with a history of coronary artery disease, mild hyperlipidemia, and hypertension. This patient twice demonstrated the severe rises in triglycerides while taking the antidepressant, *i.e.*, venlafaxine, and discontinuing the long-term use of fenofirate. The adverse drug reaction sub-committee at the hospital rated the second event as a “probable reaction” using the Naranjo nomogram, accordingly.

**Conclusions:**

This case demonstrates the risk of changes in lipid profiles while taking venlafaxine and receiving on and off fenofibrate therapy in the older adult patient with chronic kidney disease and under hemodialysis. Regular monitoring for lipid changes after starting venlafaxine is strongly advised for patients with existing risk factors.

## Background

Elevated triglycerides (TG) increase the risk for cardiac events or pancreatitis and are commonly associated with diabetes mellitus (DM), obesity, metabolic syndrome, and renal failure [1–3]. Genetics are a contributing factor in lipid disorders such as familial hypertriglycereridemia, and may also influence other hyperlipoproteinemias [4]. The use of certain medications, including estrogens, thiazide-type diuretics, isotrentinoin, antiretrovirals, and second generation antipsychotics (e.g., clozapine, olanzapine) have also been associated with hypertriglyceridemia [1, 2]. Hypertriglyceridemia is usually an asymptomatic condition and commonly is discovered only when a lipid panel is obtained.

The prescribing information for Venlafaxine extended release (ER) (Efexor®) includes a discussion about possible increasing in total cholesterol (TC) and TGs in the healthier adult patients during premarketing clinical trials [5]. A very limited number of pre-marketing trials [6–8], and no post-marketing trials which examine or report this effect could be found while performing a literature review in the PubMed database. No previous research has examined the effects of venlafaxine on TGs in patients with chronic kidney disease. We report a complicated older adult case with several risk factors for hypertriglyceridemia, and twice demonstrated the severe rises in TGs while taking the antidepressant, *i.e.*, venlafaxine, and discontinuing the long-term use of fenofirate.

## Case presentation

A 70 year-old retired male patient had the following active diseases: end-stage renal disease on hemodialysis, 3-vessel cardiac artery disease, hypertension, type-two diabetes mellitus (DM), mild hyperlipidemia, and chronic major depression. His body mass index (BMI) maintained within the normal range (e.g., 22.5 kg/m^2^ for 152 cm and 52 kg of dry weight).

The patient suffered an ischemic stroke in 2008 and received percutaneous coronary interventions in 2010 and 2013 with placement of two drug-eluting stents. The patient has been on hemodialysis three times per week since the end of 2012 and his blood pressure was controlled on medications and thyroid function was normal. The patient does not drink alcohol and his DM has been well controlled, with a hemoglobin A1C averaging 5.45% and pre-prandial blood glucose averaging 113.4 mg/dL since 2011. He had a history of low appetite and one to three times a day drank the liquid nutritional supplements formulated for dialysis patients. The patient’s medications, as of December 1st, 2015, included venlafaxine 75 mg daily and fenofibrate 200 mg every-other day (QOD), in addition to clopidogrel, carvedilol, famotidine, sennosides, levocetirizine, zopiclone, and dialysis vitamins.

Venlafaxine ER therapy was started on June 12th, 2013 to treat major depression related to coping with hemodialysis treatments. Prior to venlafaxine, mirtazapine and escitalopram were initiated and discontinued due to adverse effects of drowsiness and gastrointestinal upset, respectively. The patient had a good clinical response to venlafaxine ER soon after first use. Fenofibrate therapy was discontinued two months after venlafaxine initiation on August of 2013. That is because his TG levels were within normal limits since June 2012 and he requested to simplify his medication regimen. After four months of venlafaxine alone therapy, the patient’s TG level rose to 728 mg/dL, much higher than any time in his disease history. Afterwards, Fenofibrate 200 mg daily was given for 7 days before returning to the patient’s previous chronic dose 200 mg QOD.

**Fig. 1:**
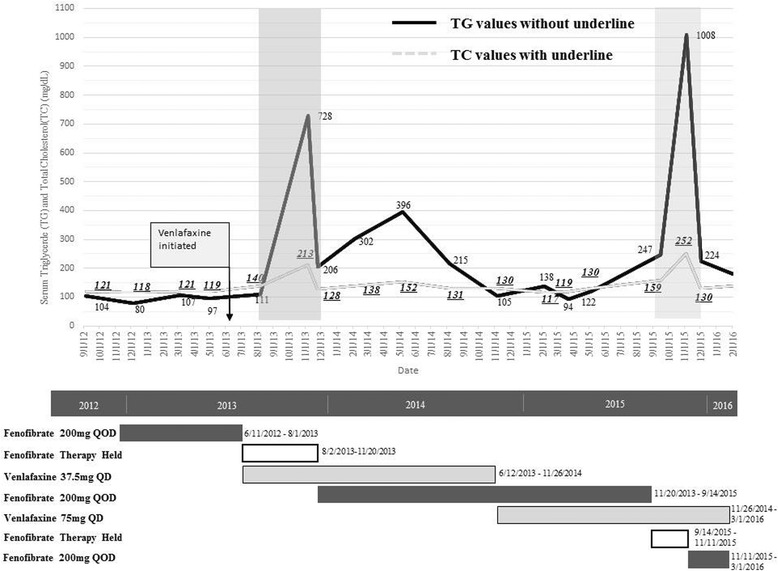
Serum triglycerides and periods without finofibrate therapy and timeline of fenofibrate and venlafaxine ER use, 2012–2016. Shaded areas mark time when patient was not prescribed finofibrate. Serum triglyceride in mg/dL to mmol/L, x0.01129

During another discontinuation of fenofibrate in order to simplify his medication regimen again in November 2015, the patient demonstrated another severe and swift rise in TG to 1,008 mg/dL while taking venlafaxine. After reinitiating fenofibrate, his TG levels dropped to 224 mg/dL. The TC levels ranged from 117 mg/dL to 280 mg/dL. The corresponding TC levels changed as TG changed over time (Fig. 1). However, the LDL-C levels were all less than 60 mg/dL and HDL ranged 32 ~ 44 mg/dL.

We compared the average TG levels of two time periods in order to account for the various confounding variables and risk factors in this patient. While this patient was on a stable hemodialysis schedule, diet, weight and all the other medications, TG levels changed as using fenofibrate alone or with venlafaxine. The patient was on fenofibrate 200 mg QOD from June 2012 to August 2013. He was also on this same dose of finofibrate while also taking venlafaxine from November 2013 to August 2015. While on fibrate therapy only, the average TG level was 97 mg/dL. While on venlafaxine in addition to identical fibrate therapy, the average TG level of 197 mg/dL was significantly higher than when on fibrate therapy alone (Fig. 1). The adverse drug reaction sub-committee of the hospital’s Pharmacy and Therapeutics Committee assessed the second event as a “possible” adverse drug event and gave a score of five on the Naranjo nomogram [9].

## Discussion

This case demonstrates an association of taking the antidepressant venlafaxine with a severe increase in TG level above 1,000 mg/dL, putting the patient at risk for pancreatitis or cardiovascular events. The drug manufacturer Pfizer was contacted to obtain information on this adverse effect. Mild rises in serum TCs and TGs were seen in pre-marketing studies [6–8]. Clinical relevant increases in serum cholesterol, defined as ≥50 mg/dL increase over baseline to a level ≥261 mg/dL, were seen in 5.3% of venlafaxine patients versus 0.0% of placebo patients in these phase three trials. Serum cholesterol labs in these premarketing studies were not specifically performed under fasting conditions and may not have had significant risk factors for dyslipidemia. Regardless, the increase in TCs and TGs is mentioned in the package insert for this drug and includes a suggestion that doctors advise patients of the risks and consider regularly measuring serum lipid levels.

Cholesterol increases have been reported in clinical trials of various antidepressants, including venlafaxine [10, 11]. The clinical relevance of these elevations or fluctuations for those antidepressants remain unstudied in patients with chronic kidney disease. The mechanism for venlafaxine’s effect on TC and TG levels is not fully understood. A study which examined the mechanism of antidepressant-induced cholesterol biosynthesis during antidepressant therapy demonstrated that this effect may be related to activation of sterol regulatory element-binding protein (SREBP) transcription factors [12]. These factors, which have been shown to increase cholesterol biosynthesis in human glial as well as peripheral cells, may contribute to the increased cholesterol levels in peripheral blood. However, venlafaxine was not included among the antidepressants evaluated in this study. Additional study has shown serotonin-norepinephrine reuptake inhibitors alter the regulation of beta-1, 2, 3 and alpha-1 receptors, which can affect the control of lipid metabolism, increasing lipolysis in adipose tissues [13].

Two previous cases have also reported a severe increase in TGs after taking venlafaxine in patients without chronic kidney disease or any co-morbidities. A 42 year-old man with social phobia and depressive disorder with no other chronic diseases or medications was placed on alprazolam and venlafaxine, which was started and tapered up to a dose of 150 mg per day [14]. Testing of the lipid panel exhibited a TG level of 1,090 mg/dL and the venlafaxine was discontinued. In a second case, a 30-year-old male with a history of depression was started on venlafaxine therapy at 150 mg a day [15]. Three months after starting therapy, the patient reported to the emergency room with severe abdominal pain and was diagnosed with acute pancreatitis. TG levels were reduced from over 3,500 to 212 mg/dL after three days of plasma exchange therapy. Further research of this potential adverse drug reaction involving antidepressant medication is needed.

## Conclusion

This case demonstrates a strong association of taking the antidepressant venlafaxine and discontinuing fenofibrate with the severe increase in TG to levels even above 1,000 mg/dL. In contrast to previous literature, this case more accurately reflects the complicated clinical situation of dialysis patients, although a genetic component cannot be ruled out. Focus should be placed on patients with existing risk factors for hyperlipidemia and receiving on and off fenofibrate therapy as these patients may be at higher risk for this adverse reaction. Steps should be taken to increase healthcare providers’ awareness of this possible effect in patients with severe kidney disease taking antidepressants. Regular monitoring for lipid changes after starting venlafaxine should be highly recommended in patients with existing risk factors. Further studies with larger populations of patients with certain targeted risk factors are also needed.
